# Genome-Wide SNP and Indel Discovery in Abaca (*Musa textilis* Née) and among Other *Musa* spp. for Abaca Genetic Resources Management

**DOI:** 10.3390/cimb45070365

**Published:** 2023-07-12

**Authors:** Cris Francis C. Barbosa, Jayson C. Asunto, Rhosener Bhea L. Koh, Daisy May C. Santos, Dapeng Zhang, Ernelea P. Cao, Leny C. Galvez

**Affiliations:** 1 Philippine Fiber Industry Development Authority (PhilFIDA), PCAF Building, Department of Agriculture (DA) Compound, Quezon City 1101, Philippinesjcasunto.philfida@gmail.com (J.C.A.); 2 Institute of Biology, College of Science, University of the Philippines Diliman, Quezon City 1101, Philippines; daconstantino@up.edu.ph (D.M.C.S.); epcao@up.edu.ph (E.P.C.); 3 National Institute of Molecular Biology and Biotechnology, University of the Philippines Diliman, Quezon City 1101, Philippines; k.rbhea@gmail.com; 4 Sustainable Perennial Crops Laboratory, United States Department of Agriculture—Agricultural Research Service, Beltsville, MD 20705, USA; dapeng.zhang@usda.gov

**Keywords:** *Musa textilis*, whole-genome resequencing, polymorphism discovery, single-nucleotide polymorphisms (SNPs), DNA insertions/deletions (InDels)

## Abstract

Abaca (*Musa textilis* Née) is an economically important fiber crop in the Philippines. Its economic potential, however, is hampered by biotic and abiotic stresses, which are exacerbated by insufficient genomic resources for varietal identification vital for crop improvement. To address these gaps, this study aimed to discover genome-wide polymorphisms among abaca cultivars and other *Musa* species and analyze their potential as genetic marker resources. This was achieved through whole-genome Illumina resequencing of abaca cultivars and variant calling using BCFtools, followed by genetic diversity and phylogenetic analyses. A total of 20,590,381 high-quality single-nucleotide polymorphisms (SNP) and DNA insertions/deletions (InDels) were mined across 16 abaca cultivars. Filtering based on linkage disequilibrium (LD) yielded 130,768 SNPs and 13,620 InDels, accounting for 0.396 ± 0.106 and 0.431 ± 0.111 of gene diversity across these cultivars. LD-pruned polymorphisms across abaca, *M. troglodytarum*, *M. acuminata* and *M. balbisiana* enabled genetic differentiation within abaca and across the four *Musa* spp. Phylogenetic analysis revealed the registered varieties Abuab and Inosa to accumulate a significant number of mutations, eliciting further studies linking mutations to their advantageous phenotypes. Overall, this study pioneered in producing marker resources in abaca based on genome-wide polymorphisms vital for varietal authentication and comparative genotyping with the more studied *Musa* spp.

## 1. Introduction

Abaca (*Musa textilis* Née) is a fiber crop endemic to the Philippines [1]. Being the center of origin of abaca, the Philippines contributes about 86.1% of the global fiber and fiber-based product requirement, generating an average annual income of USD 111.9M from 2010 to 2019 [2]. In many countries, abaca planting materials have been sourced from the Philippines, such as those planted in Borneo and islands in the Indies [3]. This makes the abaca industry an extremely important livelihood for Filipinos, wherein a total of 126,436 are abaca farmers and 160,400 hectares of land are planted [2]. Abaca is mainly harvested for its fiber, the Manila hemp. It has higher tensile strength than synthetic fibers, such as nylon and rayon, and has equivalent flexural strength with fiberglass [3,4,5]. Abaca also possesses long fiber length and high resistance to saltwater damage; thus, it is widely used for making marine cordages, paper, money, furnishings, insulators and textiles and environmentally friendly materials for automobile manufacturing [3,5,6,7,8]. A total of 170 varieties and 773 accessions have been documented in the Philippines [9,10]. To preserve genetic resources for breeding programs, germplasms are being maintained across the country by the Philippine Fiber Industry Development Authority (PhilFIDA). Varieties possessing advantageous characteristics can also be found in the wild, such as Ihalas in the Visayas, which was observed to have good tolerance to abaca diseases. There are also varieties that have been identified to be recommended for planting in particular regions, such that the fiber quality and yield obtained are optimal. In the Bicol region, *Musa* tex 51, Tinawagan Puti and Abuab are recommended for cultivation; Linawaan, Laylay and Inosa in the Visayas; and Bongolanon, Maguindanao and Tangongon in Mindanao [11]. Among these, Abuab, Inosa and Tangongon are registered under the National Seed Industry Council (NSIC) [12].

Abaca fiber production, however, is hampered by viral diseases such as bunchy top disease, mosaic disease and bract mosaic disease [13,14]. Abiotic stresses such as typhoons, severe flooding and drought also serve as important constraints to optimal abaca fiber productivity. In 2018, the abaca production was documented to decline by 8.0% as a result of El Niño, which consequently led to losses of 11.5% in fiber exports and 14.0% in abaca fiber shipment [15]. The reduced abaca fiber production is further aggravated by the absence of identified resistant varieties to both biotic and abiotic stresses. Regarding management of abaca germplasms and plantations, variety misidentification due to mislabeling during early stages of domestication, misidentifying morphologies and phenotypically variable progenies produced from seed-derived abaca plants also contribute to the retardation of the progress of the Philippine abaca industry [9]. These errors in identification of varieties have long been problems that impede the economic potential of abaca due to fluctuating qualities of fibers released in the market. Moreover, the lack of a well-curated germplasm collection produces uncertainties in the identity of plant materials used for breeding programs, leading back to the problem of the lack of highly proven resistant varieties to date. Furthermore, currently there is no information on genetic polymorphisms among abaca and other *Musa* spp. instrumental in genetic studies for advantageous or deleterious polymorphisms. Breeding for biotic and abiotic stress resistance hence requires information on abaca polymorphisms as a resource for developing genetic markers for accurate varietal identification. At the same time, information on the genetic differences among abaca and other *Musa* spp. can serve as a resource for mining of genes and polymorphisms linked to biotic and abiotic stress resistance.

To maintain the integrity and stability of the Philippine abaca industry, there is a dire need for genomic resources for the establishment of methods of identification of varieties based on their genetic nature instead of their morphologies or phenotypes. This will not only solve varietal misidentifications and labeling but will also be useful in marker-assisted selection in breeding programs, tracking movement of planting materials and authenticating varietal source of fibers released in the market. Establishing varietal identity can be best achieved using genetic markers such as single-nucleotide polymorphisms (SNPs) and DNA insertions/deletions (InDels), which are the top two most abundant DNA polymorphisms/variants in the plant genome [16,17].

SNPs are the smallest unit of genetic differences and are the most abundant sequence variations in the genomes of most organisms [18]. They have a wide distribution across the genome, with loci located within genes such as SNPs among exons, introns or promoters, and between genes [19]. The high abundance of SNPs (indicating a higher chance of finding markers linked to traits of interest), their biallelic nature leading to lower error rate in allele calls and their higher amenability to high-throughput genotyping methods such as genotyping arrays [20] and genotyping-by-sequencing (GBS; [21]) have led to the preference of using SNP markers for genetics studies [22].

InDels, which are the second most abundant genetic variation next to SNPs, are another group of markers of choice due to their codominant inheritance, wide genome distribution and multiallelic nature [18]. Compared to SNPs, InDels have higher functional consequences in organisms because they often result in frameshift mutations, disrupting the reading frames and gene expression among coding genes [18].

As of this date, only two studies [23,24] have been able to publish genetic markers for abaca genetic differentiation, which were based on banana-based simple sequence repeats (SSRs) and SNPs, respectively. These SSR markers were able to distinguish among 150 abaca accessions and revealed that the diversity within *M. textilis* is very rich, as evidenced by the calculated Shannon’s Diversity Index of 0.68 [23]. The aforementioned SNP markers were also able to distinguish among 62 abaca accessions [24]. Randomly Amplified Polymorphic DNA (RAPD) markers were also reported years earlier [25] during an international symposium. In this work, the RAPD markers were able to distinguish abaca from other *Musa* species, but the identification of varieties within *M. textilis* was not performed. Aside from varietal identification, banana-based SSR markers associated with abaca bunchy top virus (ABTV) resistance have also been used to screen potential resistance to said virus among 57 accessions [26]. Since the aforementioned markers described in these two studies were based in bananas, these findings still need further confirmation. Moreover, the genotyping accuracy of these SNPs can have limitations due to differences in the genome sequence of abaca and banana. The SNP markers were assayed using microarray technology, which is expensive and not sustainable for routine genotyping. One disadvantage of SNP arrays for variant discovery is the bias of SNP arrays for preselected SNPs that capture only a fraction of the total diversity, especially if cross-species adaptation of SNPs is attempted. The use of SNP arrays will produce genotypes only based on the preselected SNPs. In cases where a different set of variants exist in the same loci across the samples being genotyped, possible errors in allele calls could occur. This is an instance of an ascertainment bias of SNP panels [27].

To facilitate the uncovering of true genome-wide polymorphisms that will be extremely important resources for abaca varietal identification and efficient breeding programs, the declining cost and increasing accessibility of next-generation sequencing (NGS) can be taken advantage of. NGS allows the generation of huge datasets containing genome-wide variants across individuals, hence providing a highly abundant resource of genetic markers for varietal identification and comparative genotyping across individuals, populations or species [28,29]. A reference genome for a species can be first generated through high-coverage de novo whole-genome sequencing and assembly, followed by sequencing of individuals within that population. This approach is often referred to as resequencing [28].

Recently, the abaca genome has been assembled [30], thus providing a reference sequence for discovery of SNPs and InDels present in abaca itself through next-generation sequencing (NGS). With the availability of an abaca reference genome, this study aimed to mine abaca genome-wide SNPs and InDels among abaca cultivars and other *Musa* and analyze their potential as genetic marker resources through genetic diversity and phylogenetic analyses.

Specifically, this study aimed at (1) whole-genome resequencing of 11 abaca varieties and accessions, (2) mapping of sequence reads to the abaca reference genome, (3) mining and analysis of SNPs and InDels and (4) determining genetic variation and evolutionary relationships within *M. textilis* and among *Musa* spp.

## 2. Materials and Methods

### 2.1. Plant Materials

Nine varieties housed at the Mindoro Fiber Experimental Station and Seedbank (MFESS), a PhilFIDA germplasm collection located in Socorro, Oriental Mindoro, Philippines, were used for the determination of genome-wide variants in abaca in the form of SNPs and InDels. The varieties are Abuab, Inosa, Kutay-kutay, Laylay, Tangongon, Hagbayanon, Luno, Socorro and Tinawagang Puti (T-puti). The Abuab, Inosa and Tangongon varieties are among the only three abaca varieties that are currently registered under the National Seed Industry Council (NSIC) of the Philippines. NSIC registration indicates that these varieties qualified for the criteria set for commercially propagated varieties, such that they must: (1) yield at least 800 kg/hectare dried fiber, (2) have a minimum fiber recovery of 1.5%, (3) be currently planted commercially at the event of registration and (4) have been reported and described technically in reputable publications [31]. Moreover, the T-puti and Laylay are varieties recommended for planting in Bicol and Visayas areas in the Philippines [11]. Furthermore, the Kutay-kutay, Hagbayanon, Luno and Socorro varieties are varieties with good observed agronomic performance in Zamboanga. There are also other accessions present in MFESS and included in this study, such as Samoro and ‘Luno Green’; however, they are only present as a single hill. One sample from each of the 11 varieties and accessions was randomly collected at the MFESS and was flash frozen in liquid nitrogen prior to storage at −80 °C. The descriptions of these samples are listed in Table 1.

### 2.2. DNA Extraction and Sequencing

DNAs were extracted from all samples using the SEPa Plant DNA Isolation Reagent Kit (1st Base, Selangor, Malaysia). For next-generation sequencing, libraries were prepared using the Nextera DNA Library Prep Kit (Illumina, San Diego, CA, USA), and PE 150 sequencing was performed using the Illumina Hiseq 4000 platform (Illumina, San Diego, CA, USA).

### 2.3. Quality Control and Trimming of Sequence Reads

To ensure the quality of bases used for variant calling, the program Trimmomatic [34] RRID:SCR_011848 was run to remove low-quality bases and contaminating adapters. Trimming of low-quality bases was employed using the parameters LEADING:3 TRAILING:3 SLIDINGWINDOW:4:20 MINLEN:80 in Trimmomatic. To determine the quality of the sequence reads before and after quality trimming, the FastQC tool [35] RRID:SCR_014583 was utilized using the raw sequence reads and trimmed paired-end reads, respectively. MultiQC [36] RRID:SCR_014982 was then used to compile and further process the FastQC results in one report. Adapter removal was also conducted through Trimmomatic using the parameter ILLUMINACLIP:NexteraPE-PE.fa:2:30:10, which removed adapters from the Nextera DNA Library Prep Kit.

### 2.4. Mapping of Reads to the Reference Genome

The quality-filtered sequence reads were mapped to a polished version of the Abuab reference genome [30] using the BWA version 0.7.17-r1188 [37] RRID:SCR_010910. The reference genome was first indexed using the bwa index command, followed by alignment of the reads to the indexed reference genome using the bwa mem command. The product of this step is a Sequence Alignment Map (SAM) file, which is expected to contain the read alignments. Each of the SAM files was compressed into a binary format called Binary Alignment Map (BAM) using the SAMtools version 1.15.1 RRID:SCR_002105 view, collate, fixmate, sort, markdup and index commands to reduce the complexity and size of the alignment file for downstream procedures [38]. The SAMtools view command converts the complex SAM file into a simpler but unsorted BAM file. The SAMtools collate ensures that sequence reads with the same names are grouped together, while the SAMtools fixmate adds mate coordinates as well as fields for insert size [39,40]. The SAMtools sort then sorts the resulting coordinate sorted BAM file. To ensure that duplicate alignments resulting from the sequencing process are marked, the SAMtools markdup function was used. Finally, the SAMtools index command was used to enable indexing of the BAM files to make these files simpler and to allow its features to be searchable when they are used in downstream bioinformatics analysis [39,40].

The bamqc command by the Qualimap tool v.2.2.2-dev [41] RRID:SCR_001209 was then used to evaluate the generated alignments and produce statistics, such as the percentage of mapped reads, mean coverage, duplication rate, general error rate and mean mapping quality.

To serve as additional sources of polymorphisms, quality-filtered reads previously produced for the Ihalas accession [32] were included in read-mapping and variant calling steps. Sequence reads from three unidentified abaca accessions [33] deposited under the SRA accession numbers SRR8989639, SRR9696635 and SRR9850642 were included as well.

Sequence reads from *M. acuminata*, *M. balbisiana* and *M. troglodytarum* were also included in the mapping step to determine the polymorphisms present and evolutionary relationships between *M. textilis* and the other *Musa* spp. These will be important resources and information for identification of polymorphisms associated with disease resistance and advantageous agronomic traits. A summary of all sequence reads mapped to the reference genome is shown in Table 1.

### 2.5. Calling of Genome-Wide SNPs and InDels

Raw variants between the reference genome and each of the varieties were called through BCFtools version 1.15.1 RRID:SCR_005227 mpileup and call -vc commands using the sorted BAM files as input [40]. The mpileup command ensures that variants are called based on uniquely mapped reads, while the call command performs the actual calling of the SNPs/InDels from the compressed BCF file [40,42]. The -vc option was used to generate variants using the consensus caller in BCFtools [40]. The called variants were printed using the BCFtools view command, followed by initial filtering of the variants using the vcfutils.pl varFilter option. This final step produced VCF files containing the variants called between each variety and the reference genome. This variant calling pipeline was conducted for each of the abaca varieties and the additional accessions.

### 2.6. Quality Filtering of SNPs and InDels

The VCF files generated from the variant calling step were indexed and merged into one VCF file using the BCFtools commands index and merge, respectively. The SNPs and InDels were distributed into two separate VCF files using VCFtools version 0.1.16 [43] RRID:SCR_001235. Using the merged VCF as input for VCFtools, the --remove-indels option was used to generate a VCF containing SNPs only, while the --keep-only indels option was used to generate a VCF with indels only. 

To filter high-quality variants, the VCF files were used as input for VCFtools. The parameters --min-alleles 2 and --max-alleles 2 were used to select only biallelic variants, while the --minQ 40 parameter was used to select only the variants with a mapping quality higher than 40.

After quality filtering in VCFtools, the variants were further filtered using the PLINK 2.0 software (http://pngu.mgh.harvard.edu/purcell/plink/, accessed on 11 November 2022) [44] RRID:SCR_001757 executed in the R program. Highly associated variants loci were pruned to ensure absence of linkage disequilibrium (LD) between the variant positions using the parameters --indep-pairwise 50 2 0.2. LD occurs when alleles are non-randomly associated at a genetic loci pair and is observed when the observed haplotype frequencies and expected haplotype frequencies deviate, meaning the assumption that alleles at a pair of loci should have independent association was not met [45]. As a consequence, when a pair of selected variants is in LD, the information provided by one variant is redundant with the other variant. As a result, the informativeness of a set of variants containing variants in LD is reduced. This step removed pairs of variant loci within a 50 bp window with the correlation r^2^ > 0.2. The r^2^ [46] refers to the squared correlation between the presence of an allele at a locus and another allele at another locus, and is represented by the function:

$$r^{2}=\frac{{\left(pA_{i}B_{j}-pA_{i}{pB}_{j}\right)}^{2}}{pA_{i}(1-pA_{i})pB_{j}(1-pB_{j})}$$

where the ith allele frequency of the locus A and the jth allele frequency of the locus B are denoted by pA_i_ and pB_j_, respectively, and the haplotype frequency of A_i_B_j_ is represented by $pA_{i}B_{j}$ [45,47].

Pruned variants were further filtered using the parameters --geno 0.10 and --maf 0.05. The former filters out all variants with missing call rates exceeding 10% and the latter filters out all variants with minor allele frequency (MAF) below 5%, respectively. This step yielded high-quality genome-wide SNPs and InDels.

The retained high-quality polymorphisms were analyzed for their informativeness using metrics such as heterozygosity (*He*); minor allele frequency (MAF) and polymorphism information content (PIC) were also calculated. *He* provides information on the likelihood of particular loci being heterozygous; hence, *He* is a fundamental metric for estimating the genetic diversity in a population in terms of gene diversity at a locus [48,49]. *He* is calculated using the following formula:

$$\mathrm{He}=1-\sum_{i=1}^{I}{Pi}^{2}$$

wherein *I* represents the distinct number of alleles at a particular locus and *Pi* represents the frequency of allele *I* within the population [50]. MAF is a measurement of the frequency of the second most common allele in the population [51], and is calculated using the formula:

$$\mathrm{MAF}=\frac{minor\;allele\;count\;in\;the\;population}{Total\;allele\;count\;in\;the\;population}$$

Usually, MAF < 0.05 are excluded. PIC, on the other hand, is useful for measuring the polymorphism detection capability of markers within a population, and is calculated as follows:

$$\mathrm{PIC}=1-\sum_{i=1}^{n}{Pi}^{2}-{\left(\sum_{i=1}^{n}{Pj}^{2}\right)}^{2}-\sum_{i=1}^{n}{Pi}^{4}$$

where in *Pi* and *Pj* represent the frequencies of a selected marker’s *i*th and *j*th alleles, respectively [49,52]. Polymorphisms with PIC higher than 0.50 are considered very informative, while polymorphisms with PIC between 0.25–0.50 are considered somewhat informative [49]. 

### 2.7. Analysis of Genome-Wide Variation and Phylogenetic Relationships

For downstream genetic variation and phylogenetic analyses, the VCF files produced after variant pruning were preprocessed using the vcfR package [53] RRID:SCR_023453 in R. Principal component analysis (PCA) was performed using the Poppr package [54] RRID:SCR_023452 and visualized using ggplot2 [55] RRID:SCR_014601 to project the variation among the samples involved in terms of these genome-wide polymorphisms. To quantitatively assess genetic diversity, the expected heterozygosity (*He*) of each locus was calculated. Phylogenetic analysis was conducted using the Poppr package through a neighbor-joining algorithm [56] with 1000 bootstrapping. Analysis using the Poppr package was executed following the publicly available workflows and code structures [57].

## 3. Results

### 3.1. DNA Extracts and Quality of Generated Sequence Reads

The extracted DNA from the nine abaca varieties and two additional accessions, namely ‘Luno Green’ and Samoro, were of sufficient purity, with A260/A280 ratios ranging from 2.161 to 3.056; A260/A230 ratios ranging from 0.373 to 0.738; and DNA concentration ranging from 266.50 to 641.35 ng/μL (Table S1). The DNA quality and quantity were sufficient to pass the quality control procedures for next-generation sequencing.

Whole-genome resequencing through the Illumina Hiseq platform generated a total of 646,842,305 sequences, ranging from 37,019,179 to 83,388,873 sequences across the varieties, and equivalent to a total of 1,053,306,114 sequence reads (Table S2). The reads were determined to have sufficient base quality scores (Figure S1) but were highly contaminated with Nextera sequencing adapters (Figure S2). After base quality and adapter trimming with Trimmomatic, the mean base quality scores (>35) improved (Figure S3) and all adapters were successfully removed (Figure S4). This step retained 64–90% of the bases, which were then used for mapping to the reference genome.

### 3.2. Mapping Quality Statistics

**Figure 1:**
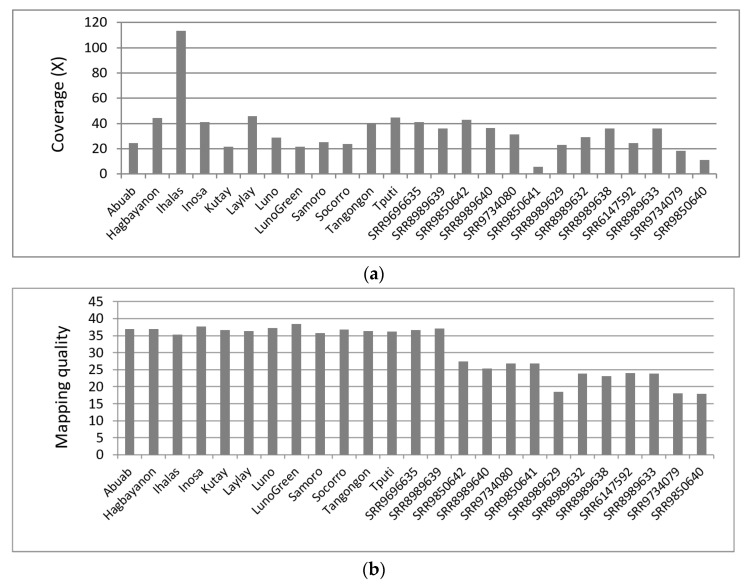
Sequencing coverage (**a**) and mapping quality evaluation (**b**) results generated through Qualimap across abaca cultivars and *Musa* accessions. The graph was generated using Microsoft Excel 2010s.

The quality-filtered sequence reads were used for mapping to the polished version (through the PILON v1.22 program) of the reference genome [30]. This step also included the mapping of *Musa* reads [33] for *M. textilis* (T genome, 2n = 2x = 20) as well as for other diploid relatives, namely, *M. acuminata* (A genome, 2n = 2x = 22), *M. balbisiana* (B genome, 2n = 2x = 22) and *M. troglodytarum* (T genome, 2n = 2x = 20) [58,59]. The BWA program was used for this step, resulting in mapping of the reads at 22–46× coverage and the calculated mean mapping qualities (MQ) having scores ranging from 35.8–38.4 across the resequenced samples (Figure 1).

Mapping of the *Musa* sequence reads [33] to the abaca reference genome revealed most accessions to map to the reference at >20× coverage except for SRR9734079 (*M. balbisiana*, 18.4×), SRR9850640 (*M. balbisiana*, 11.0×) and SRR9850641 (*M. troglodytarum*, 5.8×). Mean MQ values across these accessions also ranged between 17.9–37.1.

### 3.3. Variant Calling and Filtering Statistics

To mine and analyze the SNP and InDel polymorphisms within *M. textilis* and among *Musa* spp., variant calling was performed using the SAMtools and BCFtools programs. Variant calling produced a VCF file for polymorphisms within *M. textilis* containing 19,189,434 SNPs and 1,400,947 InDels (Table 2).

Among these, the number of high-quality (MQ > 40) and biallelic SNPs and InDels was 15,410,778 and 1,109,789, respectively. Another VCF containing polymorphisms between and among *Musa* spp. (*M. textilis*, *M. acuminata*, *M. balbisiana* and *M. troglodytarum*) was produced and contained 34,643,663 and 1,933,417 high-quality and biallelic SNPs and InDels, respectively.

Despite the huge number of polymorphisms, a total of 15,799,911 high-quality polymorphisms within *M. textilis* and 34,253,534 high-quality polymorphisms among *Musa* spp. were found to be correlated in terms of linkage (r^2^ > 0.2). To avoid capturing loci with redundant genetic information, these polymorphisms were pruned, retaining 635,945 SNPs and 84,711 InDels within *M. textilis*, and 2,130,711 SNPs and 192,835 InDels among *Musa* spp. To ensure the accuracy of downstream multivariate and phylogenetic analysis, only loci with at most 10% missing genotypes were selected, retaining 130,768 SNPs and 13,620 InDels within *M. textilis* and 31,244 SNPs and 577 InDels among *Musa* spp.

**Figure 2:**
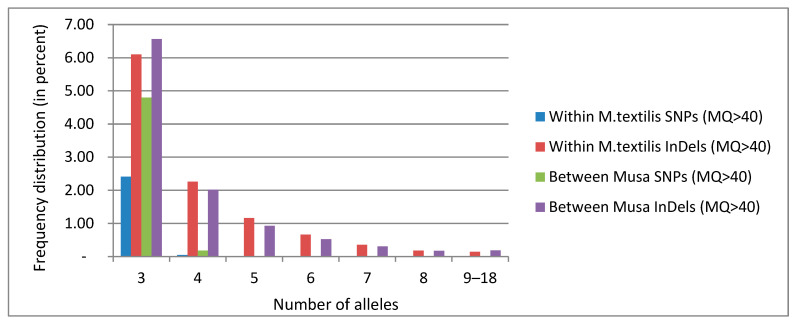
Frequency distribution of multiallelic SNPs and InDels within *M. textilis* and among *Musa* spp. The graph was generated using Microsoft Excel 2010.

A small portion of multiallelic variants within *M. textilis* was also discovered, consisting of 2.5% and 10.9% of the total SNPs and InDels, respectively (Figure 2). Among *Musa* spp., multiallelic variants consist of 5.0% and 10.7% of the total SNPs and InDels, respectively. While SNPs are known to be mostly biallelic in nature, up to four SNP alleles were detected within *M. textilis*, wherein 380,772 were triallelic and 7566 were tetra-allelic. Likewise, among *Musa* spp., up to four SNP alleles were found, comprising 1,748,160 triallelic SNPs and 142,145 tetra-allelic SNPs. For InDels, up to 18 alleles were detected, wherein 75,949 triallelic InDels (6.1% of total InDels) comprise most of the multiallelic InDels within *M. textilis*, and 142,145 triallelic InDels (6.6% of total InDels) comprising most of the multiallelic InDels among *Musa* spp. (Figure 2).

**Figure 3:**
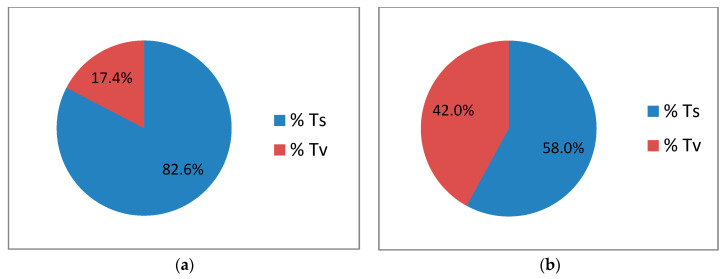
Percentage distribution of transitions and transversions among the discovered SNPs within *M. textilis* (**a**) and among *Musa* spp. (**b**). The pie chart was generated using Microsoft Excel 2010.

Calculation of transitions (Ts) and transversions (Tv) among the mined high-quality and pruned SNPs within *M. textilis* and among *Musa* spp. revealed a higher frequency of occurrence of transitions over transversions (Figure 3).

Within the *M. textilis* species, the Ts/Tv ratio was calculated to be 4.76 (Table 2). Among *Musa* spp., however, the Ts/Tv ratio was calculated to be 1.38.

To compare this dataset with existing literature, SNPs were mined within *M. acuminata* only using the accession numbers SRR8989629, SRR8989632 and SRR8989638 [33]. The mined SNPs were found to have a Ts/Tv ratio of 1.77 (Table S3), which is not far from the published findings [60] for *M. acuminata* in East Africa (Ts/Tv = 1.37).

### 3.4. Genome-Wide Variation and Phylogenetic Relationships within Musa textilis

The retained 130,768 SNPs and 13,620 InDels within *M. textilis*, and 31,244 SNPs and 577 InDels among *Musa* spp. in Table 2, represent the variants that passed the quality control procedures, and represent the genome-wide SNPs and InDels considered to be at linkage equilibrium.

**Figure 4:**
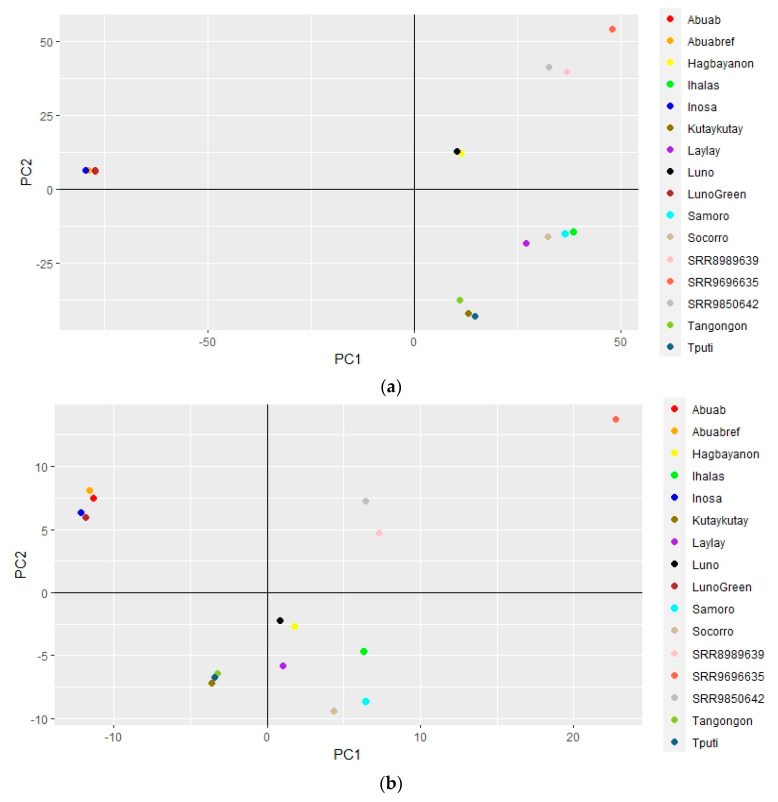
PCA plot displaying genetic variation among 16 abaca varieties and accessions in terms of their genome-wide SNPs (**a**) and InDels (**b**). These are represented by 130,768 and 13,620 LD-pruned loci, respectively, with at most 10% missing genotypes. The first and second components in the SNP PCA plot were able to explain 39.4% and 14.4% of the variance, respectively. The first and second components in the InDel PCA plot were able to explain 17.8% and 11.4% of the variance, respectively. Plots were generated using RStudio 2022.02.3.

The genome-wide variation between abaca varieties and accessions was visualized through multivariate analysis by PCA (Figure 4).

The Abuab, Inosa and ‘Luno Green’ varieties were observed to have coinciding and indistinguishable points in PCA, while the Luno and Hagbayanon varieties were observed to have partially coinciding points. The rest of the samples have points that are clearly distinguishable from each other in PCA.

Calculation of PIC, *He* and MAF across the 130,768 SNPs and 13,620 InDels revealed quantitative estimates of the genetic diversity among the abaca varieties and accessions (Table 3). In this study, the diversity among the abaca cultivars was found to be equivalent across SNPs and InDels. The mean PIC across SNPs and InDels was found to be moderate (0.312 and 0.332, respectively). Gene diversity, represented by *He*, was calculated to be higher in this study than the cross-species markers. MAF calculations were likewise found to be higher in this study than the cross-species SNPs. This indicates that the marker set in this study is more capable in detecting rare variants in populations.

**Figure 5:**
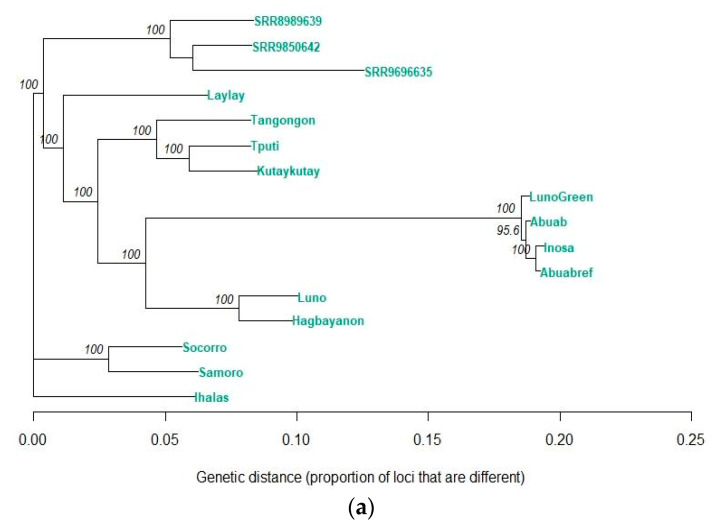

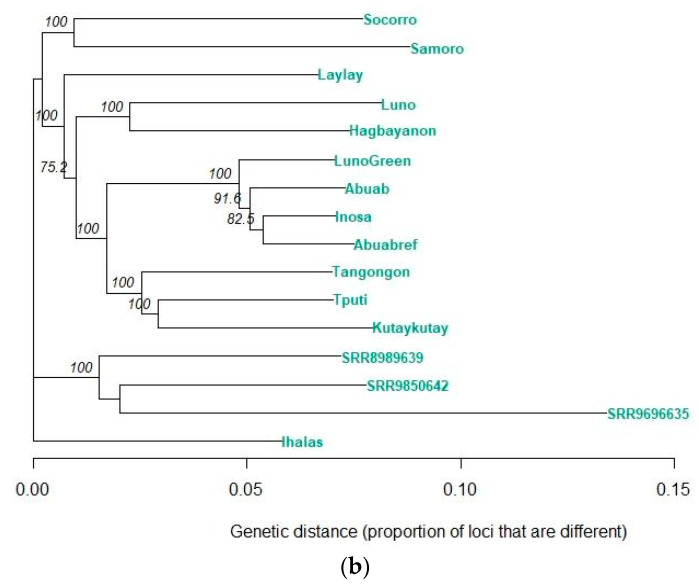
Unrooted neighbor-joining tree displaying phylogenetic relationships among 16 abaca varieties and accessions in terms of their genome-wide SNPs (**a**) and InDels (**b**). These are represented by 130,768 and 13,620 LD-pruned loci, respectively, with at most 10% missing genotypes. Values on nodes represent bootstrap support out of 1000 NJ bootstrap sampling using Hamming distance for genetic distance calculation. The accessions SRR9696635, SRR8989639 and SRR9850642 were not identified in terms of their varietal identity [33], and hence were labeled as accession numbers. Trees were generated using RStudio 2022.02.3.

A neighbor-joining (NJ) tree was also generated using Hamming distance (through the bitwise.dist function) for calculation of pairwise genetic distances, i.e., the proportion of dissimilar loci between varieties/accessions [61] (Figure 5).

The NJ tree generated using the genetic distances generated for the SNP genotypes grouped the varieties and accessions into three main monophyletic groups. The accessions [33], namely, SRR9696635, SRR8989639 and SRR9850642, as well as the abaca varieties in the current study, namely Laylay, Tangongon, Tputi, Kutaykutay, Hagbayanon, Luno, ‘Luno Green’, Abuab and Inosa, were grouped within the first main clade. The Samoro and Socorro varieties made up the second main clade, and the Ihalas accession is the lone constituent of the third clade (Figure 5a). Within the first clade, the Abuab, Inosa and ‘Luno Green’ varieties clustered together and were deeply separated from the other varieties and accessions. The branch length that immediately precedes their divergence was found to be significantly longer than the other varieties. On the other hand, the Socorro, Samoro and Ihalas varieties experienced the fewest mutations among the other varieties and accessions.

### 3.5. Genome-Wide Variation and Phylogenetic Relationships among Musa Species

**Figure 6:**
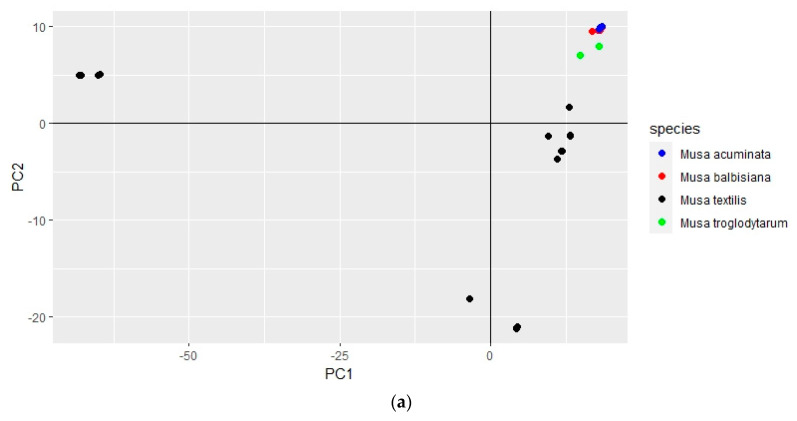

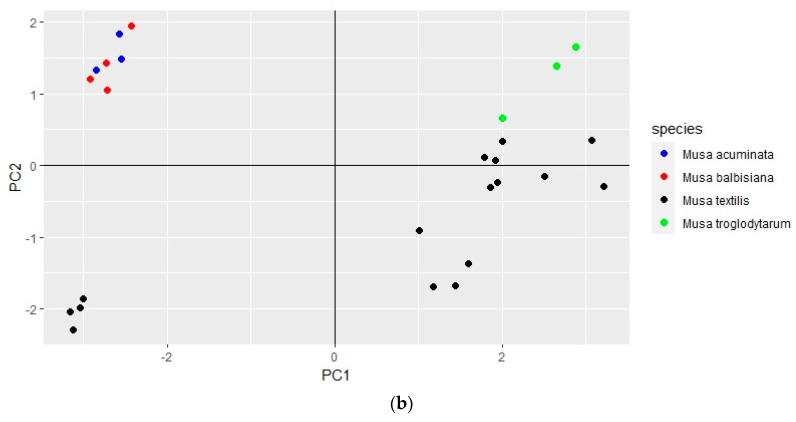
PCA plot displaying genetic variation among *Musa* species in terms of their genome-wide SNPs (**a**) and InDels (**b**). These are represented by 31,244 and 577 LD-pruned loci, respectively, with at most 10% missing genotypes. The first and second PCA components in the SNP PCA plot were able to explain 69.3% and 9.4% of the variance. The first and second PCA components in the InDel PCA plot were able to explain 27.0% and 7.9% of the variance. Species labels ‘Musa balbisiana’, ‘Musa troglodytarum’, ‘Musa acuminata’ and ‘Musa *textilis*’ represent the species *Musa balbisiana*, *M. troglodytarum*, *M. acuminata* and *M. textilis*, respectively. Plots were generated using RStudio 2022.02.3.

PCA of the SNP and InDel genotypes using the VCF file containing polymorphisms among *Musa* spp. distinguished *M. textilis* and *M. troglodytarum* species, and distinguished each of the *Musa* accessions, indicating the resolving power of InDels for genetic differentiation (Figure 6).

The *M. balbisiana* and *M. acuminata* accessions, however, did not form separate clusters. Nevertheless, the SNP and InDels were able to group the accessions into *M. textilis*, *M. troglodytarum* and *M. acuminata*/*balbisiana*. In addition, PCA analysis of the InDel genotypes also enabled separation among all samples involved (Figure S5).

**Figure 7:**
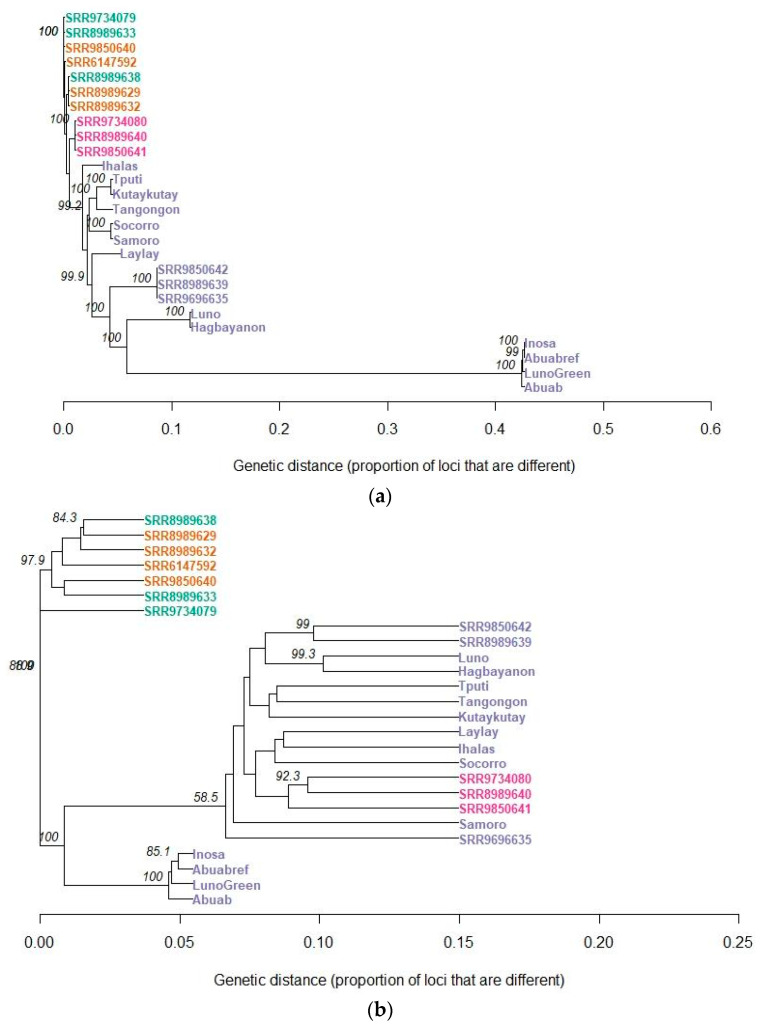
Rooted unweighted pair group method with arithmetic mean (UPGMA) tree displaying phylogenetic relationships among *Musa* accessions in terms of their genome-wide SNPs (**a**) and InDels (**b**). These are represented by 31,244 and 577 LD-pruned loci, respectively, with at most 10% missing genotypes. Values on nodes represent bootstrap support out of 1000 UPGMA bootstrap sampling using Hamming distance for genetic distance calculation. *M. textilis* accessions are labeled in purple, *M. troglodytarum* accessions are labeled in pink, *M. acuminata* accessions are labeled in green and *M. balbisiana* accessions are labeled in orange. For both trees, the SRR9734079 *M. acuminata* accession was used as outgroup from where the trees were rooted. Trees were generated using RStudio 2022.02.3.

Like the polymorphisms within *M. textilis*, genetic distances were likewise calculated among all *Musa* accessions involved in this study (Table S5). The rooted unweighted pair group method with arithmetic mean (UPGMA) analysis using the genetic distances generated for the SNP genotypes (Figure 7a) showed that all abaca varieties and accessions formed a monophyletic group and are closely related with *M. troglodytarum* among other *Musa* spp. UPGMA analyses of the InDel genotypes (Figure 7b), however, showed clustering of the *M. troglodytarum* accessions within the *M. textilis* clade, particularly the subclade containing SRR9850642, SRR8989639, Luno, Hagbayanon, Tputi, Tangongon, Kutaykutay, Laylay, Ihalas, Socorro, Samoro and SRR9696635. This subclade is distinct from the other subclade that contains the other varieties of *M. textilis*, which includes Inosa, Abuab and ‘Luno Green’.

### 3.6. Genetic Characterization of the Musa Accessions

**Figure 8:**
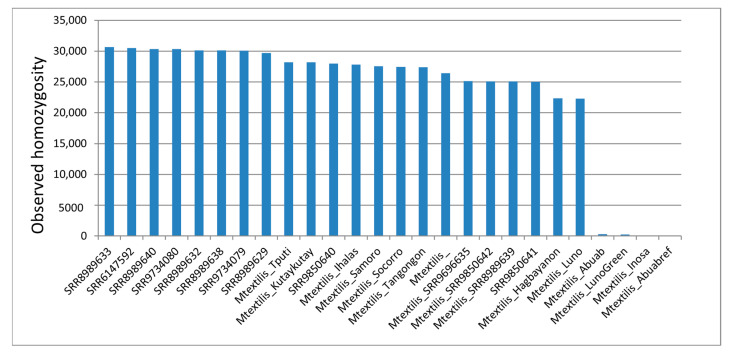
Graphical comparison of homozygosity statistics per individual (variety and accession) based on genome-wide SNPs. Labels starting with ‘Mbalbisiana’, ‘Mtroglodytarum’, ‘Macuminata’ and ‘Mtextilis’ represent varieties/accessions under the *Musa balbisiana*, *M. troglodytarum*, *M. acuminata* and *M. textilis*, respectively. The specific varieties/accessions are indicated following the aforementioned labels. The pie chart was generated using Microsoft Excel 2010.

Homozygosity statistics calculated through the VCFtools program across all accessions revealed Abuab, Inosa and ‘Luno Green’ to have a significantly low homozygosity, indicating a significantly high heterozygosity among all accessions (Figure 8).

The inbreeding coefficient calculations further support these findings, wherein Abuab, Inosa and ‘Luno Green’ were ranked last and highly negative in terms of inbreeding coefficient (F) (Table 4).

## 4. Discussion

The persisting problems of destructive abaca diseases and abiotic stresses and the slow development of abaca breeding programs for disease resistance and climate resilience, aggravated by confusions in the true identity of abaca varieties and unavailability of abaca genome-based genetic marker resources, impede the Philippine abaca industry.

The whole-genome resequencing approach is a useful method for identification of genome-wide polymorphisms vital for plant genetic resources management [62]. While a high read depth would provide assurance in genotype calling accuracy, applying this for a large number of samples can be expensive. A cost-effective solution in mining true polymorphisms is the low-depth resequencing of several individuals, which is based on the information that true variants are expected to appear multiple times upon mapping of sequence reads to the reference [28]. In *Musa*, this resequencing approach has been previously employed [63] for detection of hidden diversity within the *Musa itinerans* species by mining genome-wide variation using sequencing data with 15.5× coverage. This approach was employed in another study (through resequencing of at least 20× coverage) for multiple *Musa* species to generate and analyze genomic resources for polymorphism detection usable for studying *Musa* evolution, diversity and breeding [33].

The current study utilized the resequencing approach in an attempt to capture genetic diversity and relationships among abaca cultivars and among *Musa* spp. That are important for addressing the scarcity of genetic information and resources vital for efficient abaca breeding programs. Specifically, these are the lack of abaca genome-based genetic markers usable for accurate identification of abaca varieties used for mass cultivation and as breeding materials, and the lack of genetic marker resources for identification of polymorphisms across *Musa* spp. that are associated with agronomically and economically important traits. 

The first steps undertaken were the resequencing of 11 abaca varieties and accessions at a minimum of 20× coverage and mapping the reads to the abaca reference genome [30]. The calculated base quality scores (>35) indicate high confidence in the base calls from the sequencing (Figure S3). The calculated coverages (22–46×) matched the coverage used in a related study [33], hence indicating the sufficiency of the reads for variant calling using the abaca reference genome previously assembled at high (65×) coverage [30] (Figure 1). Using the MQ formula [64], the MQ scores among the resequenced abaca samples (35.8–38.4) indicate a very low 0.014–0.026% probability that the reads were incorrectly mapped to the reference genome. The reads, therefore, were uniquely mapped to the reference genome, indicating the high accuracy of the mapping step (Figure 1).

Mapping of the published *Musa* sequence reads [33] to the abaca reference genome [30] produced similar coverages (except for three accessions) and lower MQ values. Calculation of the mismapping probability reveals 0.02–1.62% probability that the reads were incorrectly mapped. Hence, even though the MQ values for these accessions were lower than the resequenced abaca samples and the coverage obtained for SRR9850640 and SRR9850641 are significantly lower than the other accessions, their mapping accuracy to the abaca reference genome is nevertheless satisfactory.

Mining of genome-wide SNPs and InDels was achieved using a bioinformatics pipeline featuring SAMtools and BCFtools. This step produced 20,590,381 variants within *M. textilis*, consisting of 93.2% SNPs and 6.8% InDels, and 47,580,541 variants among *M. textilis*, *M. acuminata*, *M. balbisiana* and *M. troglodytarum*, consisting of 94.8% SNPs and 5.2% InDels. This number of polymorphisms among *Musa* spp. is significantly higher compared to those within *M. textilis* polymorphisms owing to the higher expected interspecific (i.e., between-species) genetic diversity than infraspecific (i.e., within-species) genetic diversity. Among the mined polymorphisms, the high-quality (MQ > 40) SNPs and InDels were selected, and accounted for 80.3% and 79.2% of their respective total number within *M. textilis*, and 76.7% and 78.3% of their respective total number among the *Musa* spp. Even though mean MQ values around 17 were regarded as acceptable based on the equivalent mismapping probability, filtering of individual polymorphisms was based on more stringent MQ values to ensure optimal accuracy of these mined polymorphisms.

Since the majority of the polymorphisms have MQ above 40, this indicates that most of the genomic loci have accurately mapped reads. The accuracy of the identified polymorphisms is further assured by the fact that most of the polymorphisms within *M. textilis* have MQ values within the range of 57.5–60.0 (Figure S6). This MQ range indicates a 1/100,000 probability that the reads were incorrectly mapped, therefore giving information that the reads were indeed uniquely mapped to the reference genome [28,63].

While both SNPs and InDels are predominantly biallelic, multiallelic loci were also identified. For SNPs, the percentage of multiallelic loci is justifiably small (~2.3–4.7% of the total number of SNPs), owing to their supposedly biallelic nature [65]. The percentage of multiallelic SNPs is notably higher among the four *Musa* spp. than within *M. textilis*. SNP loci with greater than two alleles, such as those stated for triallelic loci [66], enable better genetic discrimination than biallelic loci. This highlights the possible role of multiallelic SNPs in genetic and phenotypic differentiation among the *Musa* spp. For InDels, which are naturally multiallelic, a total of 18 alleles were identified and have similar proportions within *M. textilis* and among *Musa* spp. Even though a high number of alleles were identified, setting the mapping quality threshold to MQ > 40 ensures accuracy of these mined InDels.

Further analysis of the SNPs revealed the transition/transversion ratio to be higher within *M. textilis* (4.76) than among *Musa* spp. (1.38). Transitions are single-base mutations resulting in the nucleotide with the same number of rings as the wild type (e.g., purine to purine mutation), while transversions occur as a result of mutation of the wildtype nucleotide base into a base with different number of rings (e.g., purine to pyrimidine mutation) [67]. Since transversions have a higher potential in altering the primary structure of encoded proteins and are more likely to alter the shape of the DNA backbone, transversions have higher impacts on gene regulatory elements and consequentially on gene expression [67]. Since SNPs have lower Ts/Tv ratios among *Musa* spp., there is a higher rate of transversion; this could be another source of phenotypic differences among the *Musa* spp.

The current study thus suggests that the higher percentage of multiallelic loci and higher transversion rate among the four *Musa* spp. than within the *M. textilis* species contributed to the genetic differentiation across the four *Musa* spp., and these findings can serve as a basis for further research on the phenotypic implications of these genetic variations in *Musa*.

Multivariate analysis (through PCA) of the high-quality polymorphisms that were pruned for linkage disequilibrium revealed genetic variation within *M. textilis* and among *Musa* spp. PCA of the SNP genotypes within *M. textilis* revealed a high similarity between the Abuab reference and the resequenced Abuab variety. It also enabled differentiation among abaca varieties based on their SNP profiles, except for two sets of varieties in which their SNP profiles were projected by coinciding points (Figure 4a). The overlapping points by Abuab, Inosa and ‘Luno Green’ varieties indicate their high genetic similarity in terms of SNPs. This provides evidence that the uncharacteristic morphology of the ‘Luno Green’ accession is a result of mislabeling in the field as a Luno variety. This is supported by initial morphological characterization studies by PhilFIDA revealing some Inosa collections possess the green male flower coloration observed for ‘Luno Green’. Using the genome-wide InDel genotypes, PCA resulted in improved resolution among the abaca samples, as represented by the absence of coinciding points (Figure 4b). Calculation and comparison of mean PIC, however, show equivalent mean PIC between the InDel loci (0.33 ± 0.08) and SNP loci (0.31 ± 0.07) (Table 3), both of which display moderate polymorphism detection capability.

Calculation of *He* for each locus provided information on genetic diversity among the *M. textilis* varieties and accession. In comparison with the only published literature on abaca SNPs, i.e., the banana genome-based SNP markers cross-species adapted into abaca [18], this current study produced a significantly higher *He* than the cross-species markers. This therefore indicates the higher capability of the abaca genome-based SNPs mined in this study to capture genetic variation within the *M. textilis* species compared to the cross-species markers. The mean MAF calculations, which are also genetic diversity metrics since they measure the frequency of the second most common allele in the population [51], were also found to be higher across the SNP loci in the current study than those in the cross-species adapted SNPs [24]. These metrics therefore indicate sufficient capability of the polymorphism markers mined in this study to capture genetic diversity within the *M. textilis* species, and thus are usable for genetic differentiation applications such as varietal identification.

To elucidate phylogenetic relationships within *M. textilis*, a neighbor-joining tree generation approach was conducted. The calculated pairwise genetic distances among the abaca varieties and accessions are listed in Table S4. Since the branch length indicates the degree of evolutionary divergence such as the number of substitutions along a particular branch, NJ analysis using Hamming distances revealed extensive mutations experienced by Abuab, Inosa and ‘Luno Green’ compared to the rest of the varieties and accessions, as evidenced by their long branch length and deep separation from the rest of the varieties and accessions [68]. This is supported by the PCA plot in Figure 4a, wherein these varieties are closely clustered with each other and away from the other varieties and accessions. The Abuab reference genome was found to be 0.7 ± 0.1% dissimilar (on average) with Inosa and ‘Luno Green’ in terms of the genome-wide SNPs and 4.2 ± 0.3% dissimilar in terms of the genome-wide InDels. In contrast, the Abuab reference genome was calculated to be 18.1 ± 6.3% and 14.2 ± 3.1% dissimilar with the rest of the abaca varieties and accessions in terms of the genome-wide SNPs and InDels, respectively. The extensive mutations experienced by the Abuab and Inosa varieties are explained by their extensive domestication due to their popularity as commercial varieties. Since seeds have been rampantly used as planting materials during the 1900s and the abaca reproductive cycle consists of alternating emergence of male and female flowers (thereby preventing selfing events), genetic variability arising from continuous cross-pollination within the population most likely occurred [9]. In contrast, the Socorro, Samoro and Ihalas varieties were observed to form a separate cluster away from the rest of the abaca samples and shared the oldest common ancestor. The separate clades formed by these three abaca varieties are explained by genetic isolation by distance (IBD). IBD occurs due to the limit in gene flow as a result of geographical isolation [69]. The Socorro and Samoro varieties are popularly planted only in the Mindoro province, while the Ihalas variety is a wild variety known to exist only in the Leyte province.

Both the NJ tree for the SNP and InDel loci clustered together the pairs Samoro and Socorro, Luno and Hagbayanon and Tangongon and Tputi (Figure 5b). The branch lengths within these pairs, however, have higher length differences observed in the InDel NJ tree compared to those observed in the SNP tree, indicating a higher degree of separation within these pairs of varieties due to differences in the number of InDel mutations, which typically arise from polymerase slippage and unequal meiotic crossing-over [70]. To establish phylogenetic relationships among *Musa* spp., UPGMA analysis was performed. Within *M. textilis*, the Ihalas was observed to be the abaca accession closest to *M. troglodytarum*, and the clusters that were formed were in agreement with Figure 5a. Since Ihalas was reported by farmers in Leyte to display tolerance to abaca viral disease, its close relationship with a banana species could have contributed to its disease tolerance. Banana species, especially *M. balbisiana*, were widely documented to possess degrees of tolerance to viral diseases, e.g., [71]. These data, hence, support the use of Ihalas as a potential source of disease resistance genes and as a candidate for intraspecific hybridization. 

Since SNPs are the most abundant genetic variation and are significantly more abundant than InDels (Table 2), the phylogenetic clustering based on SNPs observed in Figure 7a indicates the phylogenetic relationships across *Musa* spp., and then the insertion/deletion events in Figure 7b may likely contribute to enhancement of genetic diversity among *Musa* spp. and within *M. textilis*. These findings, together with the homozygosity data in Figure 8 and inbreeding coefficient calculations in Table 4, complement the NJ analysis results in Figure 5a, further supporting that the Abuab and Inosa varieties resulted from abaca being highly cross-pollinated due to alternating emergence of male and female flowers and extensive domestication exacerbated by the utilization of seeds as planting materials during the early times [9]. The results, therefore, indicate that the Abuab, Inosa and ‘Luno Green’ varieties have accumulated the most mutations among varieties and accessions in this study, and could be interesting sources of possibly advantageous mutations leading to disease resistance, abiotic stress resistance (including climate change adaptability) and good agronomic traits. Since Abuab and Inosa are NSIC-registered varieties, the advantageous characteristics of these varieties, hence, could have arisen from advantageous phenotypes caused by accumulation of advantageous mutations over time.

Overall, this study successfully mined genome-wide SNPs and InDels in abaca, which served as highly important resources for genetic marker discovery and downstream applications in genetic resources management such as varietal identification. Their potential as genetic marker resources is exemplified by their capability to detect variation within the *M. textilis* species and between *Musa textilis*, *M. acuminata*, *M. balbisiana* and *M. troglodytarum*. This is further supported by the elucidated genome-wide variations, genetic diversity and evolutionary relationships among abaca varieties and other *Musa* spp. While markers for abaca genetics studies were developed in the past few years, these markers were based on the banana genome. This study, hence, is the first report on genome-wide polymorphisms across abaca varieties based on the genome of abaca itself. Consequently, this is a pioneering work elucidating the genome-wide variation and evolutionary information across abaca varieties and among the four *Musa* species.

The genetic markers with sufficient diversity and polymorphism detection capability generated in this study will be impactful for the development of abaca genome-based assays for routine identification of abaca varieties and accessions. The mined polymorphisms among abaca and the more extensively studied banana will be important resources for identifying genes (through comparative genotyping) that may be important for abaca crop improvement and climate change adaptability. Altogether, these will lead to an efficient and effective abaca breeding program that will highly benefit the Philippine abaca industry and its global stakeholders. 

For future studies, more phenotypically associated markers will need to be mined from the between-*Musa* polymorphisms identified in this study to enable the discovery of polymorphisms in the widely studied banana species that are linked to important traits. Since phylogenetic analysis using the genome-wide SNPs revealed the Abuab and Inosa varieties to have experienced a significant number of mutations, further characterization studies of these two varieties are recommended. This will identify agronomically important traits that resulted from accumulation of possibly advantageous characteristics.

## Figures and Tables

**Table 1:** Abaca and *Musa* spp. accessions involved in the study.

Accession	Species	Description	Source of Sequence Reads	Accession Number
Abuabref	*M. textilis*	Reference genome	[30]	N/A
Abuab	*M. textilis*	Commercial abaca variety	This study	SRR22906090
Hagbayanon	*M. textilis*	Commercial abaca variety	This study	SRR22906084
Ihalas	*M. textilis*	Wild abaca accession	[32]	N/A
Inosa	*M. textilis*	Commercial abaca variety	This study	SRR22906089
Kutay	*M. textilis*	Commercial abaca variety	This study	SRR22906087
Laylay	*M. textilis*	Commercial abaca variety	This study	SRR22906086
Luno	*M. textilis*	Commercial abaca variety	This study	SRR22906083
LunoGreen	*M. textilis*	Luno with green inflorescence	This study	SRR22906080
Samoro	*M. textilis*	Present as a single hill in MFESS	This study	SRR22906088
Socorro	*M. textilis*	Commercial abaca variety	This study	SRR22906082
Tangongon	*M. textilis*	Commercial abaca variety	This study	SRR22906085
Tputi	*M. textilis*	Commercial abaca variety	This study	SRR22906081
Unknown cultivar	*M. textilis*	Abaca accession	[33]	SRR9696635
Unknown cultivar	*M. textilis*	Abaca accession	[33]	SRR8989639
Unknown cultivar	*M. textilis*	Abaca accession	[33]	SRR9850642
Banana	*M. acuminata*	Wild banana accession	[33]	SRR8989638
Banana	*M. acuminata*	Wild banana accession	[33]	SRR8989629
Banana	*M. acuminata*	Wild banana accession	[33]	SRR8989632
Banana	*M. balbisiana*	Wild banana accession	[33]	SRR8989633
Banana	*M. balbisiana*	Wild banana accession	[33]	SRR9734079
Banana	*M. balbisiana*	Wild banana accession	[33]	SRR6147592
Banana	*M. balbisiana*	Wild banana accession	[33]	SRR9850640
Banana	*M. troglodytarum*	Wild banana accession	[33]	SRR8989640
Banana	*M. troglodytarum*	Wild banana accession	[33]	SRR9734080
Banana	*M. troglodytarum*	Wild banana accession	[33]	SRR9850641

**Table 2:** Variant calling and filtering statistics.

	Within *M. textilis*		Among *Musa* spp.	
	SNPs	InDels	SNPs	InDels
Total	19,189,434	1,400,947	42,647,249	2,466,646
High quality (MQ > 40), biallelic	15,410,778	1,109,789	34,643,663	1,933,417
High quality (MQ > 40), multiallelic	388,338	135,230	1,814,554	2,31,670
Number of transitions (Ts)	108,067	N/A	18,106	N/A
Number of transversions (Tv)	22,701	N/A	13,138	N/A
Ts/Tv ratio	4.76	N/A	1.38	N/A
Retained after LD pruning	635,945	84,711	2,130,711	192,835
Number of loci with at most 10% missing genotypes	130,768	13,620	31,244	577

**Table 3:** Mean polymorphism information content, expected heterozygosity, minor allele frequency and nucleotide diversity across SNPs and InDels scored on 16 *M. textilis* varieties and accessions.

Diversity Metrics	This Study		[24]
	SNPs	InDels	SNPs
PIC	0.312 ± 0.068	0.332 ± 0.076	-
*He*	0.396 ± 0.106	0.431 ± 0.111	0.281 ± 0.135
MAF	0.310 ± 0.126	0.362 ± 0.124	0.196 ± 0.132

**Table 4:** Inbreeding coefficient (F) statistics across *Musa* accessions.

Accession/Variety	Species	N_SITES	F
SRR8989629	*M. acuminata*	30,123	0.93086
SRR6147592	*M. balbisiana*	30,963	0.928
SRR9850640	*M. balbisiana*	28,416	0.92696
SRR9734079	*M. balbisiana*	30,554	0.9252
SRR8989632	*M. acuminata*	30,643	0.91983
SRR8989633	*M. balbisiana*	31,192	0.91946
SRR8989638	*M. acuminata*	30,648	0.91891
SRR9850641	*M. troglodytarum*	25,747	0.86544
SRR8989640	*M. troglodytarum*	31,205	0.8618
SRR9734080	*M. troglodytarum*	31,204	0.85997
Ihalas	*M. textilis*	30,374	0.57407
Kutaykutay	*M. textilis*	31,079	0.53542
Tputi	*M. textilis*	31,165	0.52615
Samoro	*M. textilis*	30,835	0.46154
Socorro	*M. textilis*	30,819	0.45137
Tangongon	*M. textilis*	31,072	0.40478
Laylay	*M. textilis*	30,220	0.35956
SRR9696635	*M. textilis*	31,032	0.04446
SRR8989639	*M. textilis*	31,185	0.02363
SRR9850642	*M. textilis*	31,214	0.02259
Luno	*M. textilis*	30,726	−0.37979
Hagbayanon	*M. textilis*	30,791	−0.38391
Abuab	*M. textilis*	29,625	−3.893
LunoGreen	*M. textilis*	29,761	−3.90269
Inosa	*M. textilis*	31,039	−3.97664
Abuabref	*M. textilis*	31,180	−3.99309

## Data Availability

The sequence reads generated in the current study are available in the NCBI repository (BioProject ID PRJNA916350). The following accession numbers were generated for the paired-end sequence reads produced for each sample: SRR22906090 (Abuab), SRR22906084 (Hagbayanon), SRR22906089 (Inosa), SRR22906087 (Kutaykutay), SRR22906086 (Laylay), SRR22906083 (Luno), SRR22906080 (‘Luno Green’), SRR22906088 (Samoro), SRR22906082 (Socorro), SRR22906085 (Tangongon) and SRR22906081 (T.puti). The generated VCF files are stored and are accessible through the Digital Object Identifier DOI: 10.17632/27kywgfynh.1 stored under Mendeley Data.

## Supplementary Materials

The following supporting information can be downloaded at: https://www.mdpi.com/article/10.3390/cimb45070365/s1.
