# Supplementary material for: Genome-Wide SNP and Indel Discovery in Abaca (Musa textilis Née) and among Other Musa spp. for Abaca Genetic Resources Management
Source: Curr Issues Mol Biol. 2023 Jul 12;45(7):5776–97. doi: 10.3390/cimb45070365 (PMC10377871; doi:10.3390/cimb45070365)
Supplement: Supplementary file 1 [file cimb-45-00365-s001.zip › List of Supplementary Materials.pdf]

SUPPLEMENTARY DATA

Figure S1. Mean Phred quality scores of the sequence reads generated from abaca varieties and *Musa* accessions before low-quality trimming.

Figure S2. Mean Phred quality scores of the sequence reads generated from abaca varieties and *Musa* accessions before adapter trimming.

Figure S3. Mean Phred quality scores of the sequence reads generated from abaca varieties and *Musa* accessions after low-quality trimming.

Figure S4. Mean Phred quality scores of the sequence reads generated from abaca varieties and *Musa* accessions after adapter trimming.

Figure S5. PCA plot displaying genetic variation among *Musa* accessions in terms of their genome-wide InDels.

Figure S6. Histogram generated through Qualimap representing the quality of mapping of *M. textilis* reads to the reference genome.

Table S1. DNA quality and quality assessment by spectrophotometry.

Table S2. Raw sequencing and mapping statistics generated for *M. textilis* varieties/ accessions and *Musa* accessions.

Table S3. Summary of transitions and transversions found across three *Musa acuminata* accessions SRR8989629, SRR8989632 and SRR8989638.

Table S4. Pairwise genetic distances (Hamming distances) calculated among abaca varieties and accessions in terms of the genome-wide SNPs (below gray diagonal) and InDels (above gray diagonal).

Table S5. Pairwise genetic distances (Hamming distances) calculated among *Musa* accessions in terms of the genome-wide SNPs (below gray diagonal) and InDels (above gray diagonal).
