# Supplementary figures and images for: Genome-Wide SNP and Indel Discovery in Abaca (Musa textilis Née) and among Other Musa spp. for Abaca Genetic Resources Management
Source: Curr Issues Mol Biol. 2023 Jul 12;45(7):5776–97. doi: 10.3390/cimb45070365 (PMC10377871; doi:10.3390/cimb45070365)

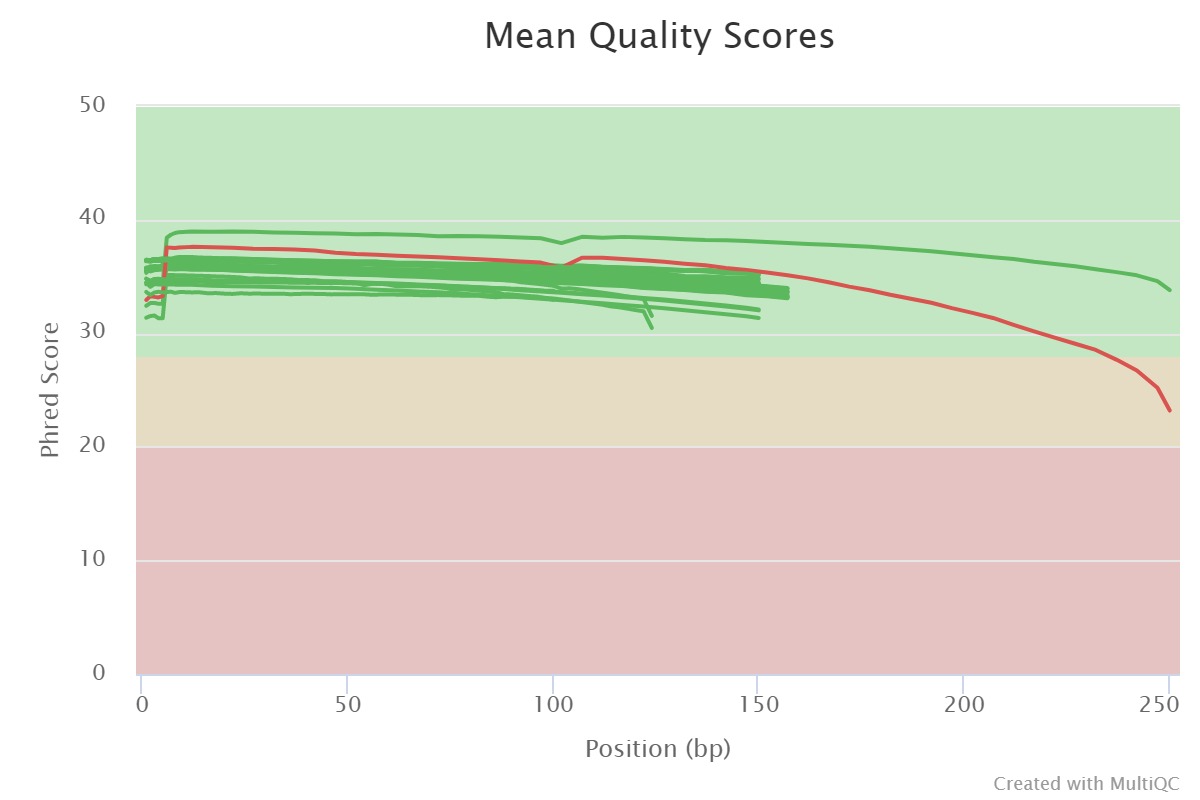

Supplement: Supplementary file 1 [file cimb-45-00365-s001.zip › Figure S1.jpeg]

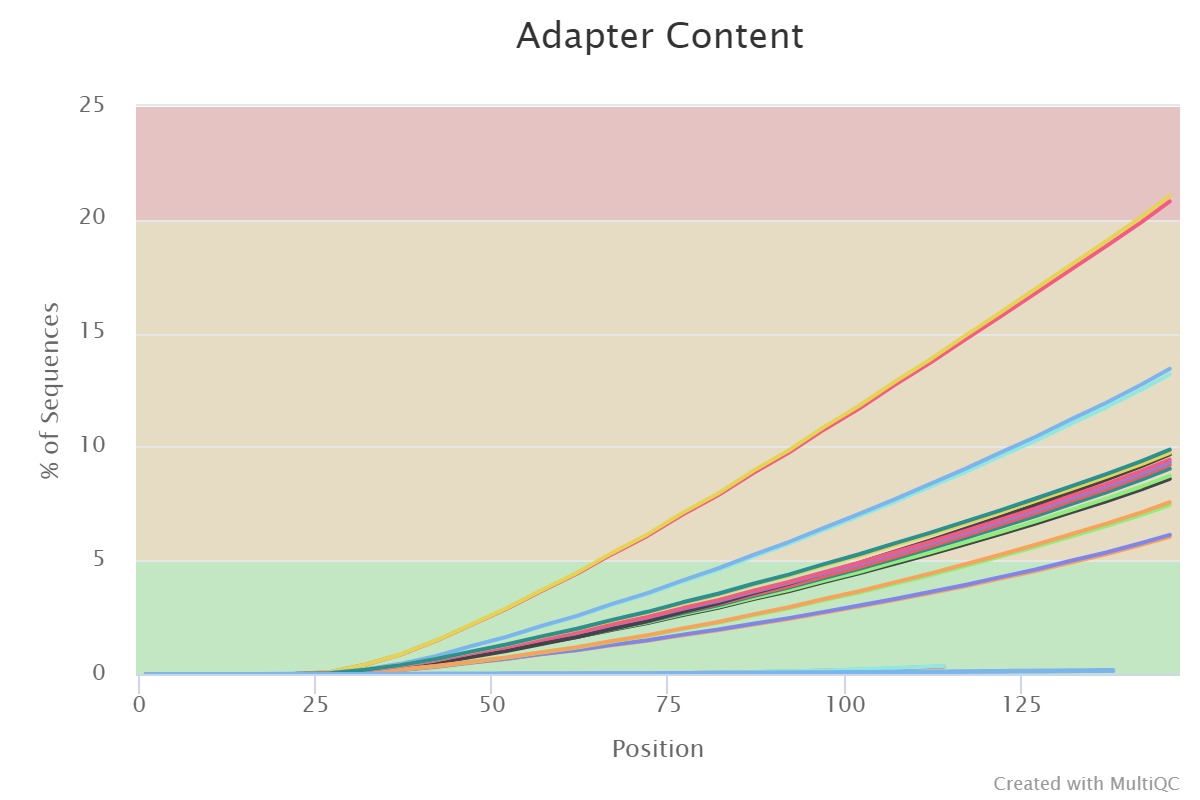

Supplement: Supplementary file 1 [file cimb-45-00365-s001.zip › Figure S2.jpeg]

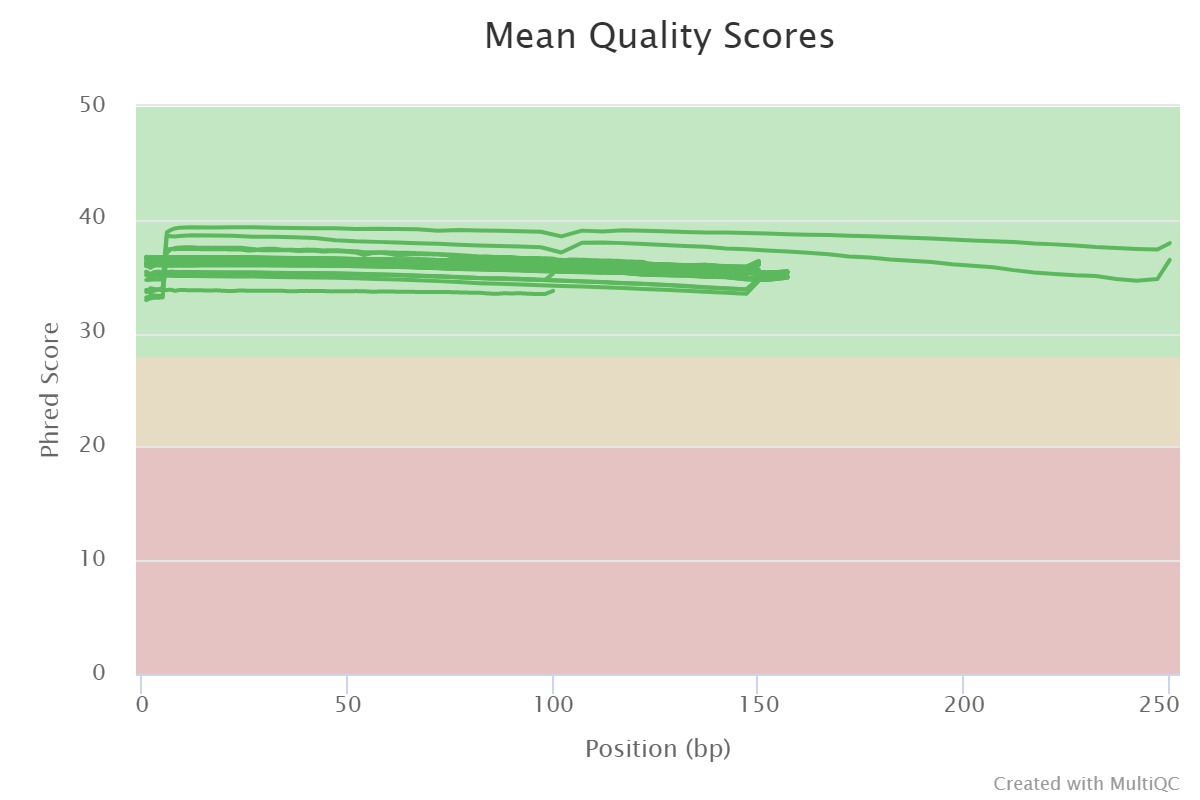

Supplement: Supplementary file 1 [file cimb-45-00365-s001.zip › Figure S3.jpeg]

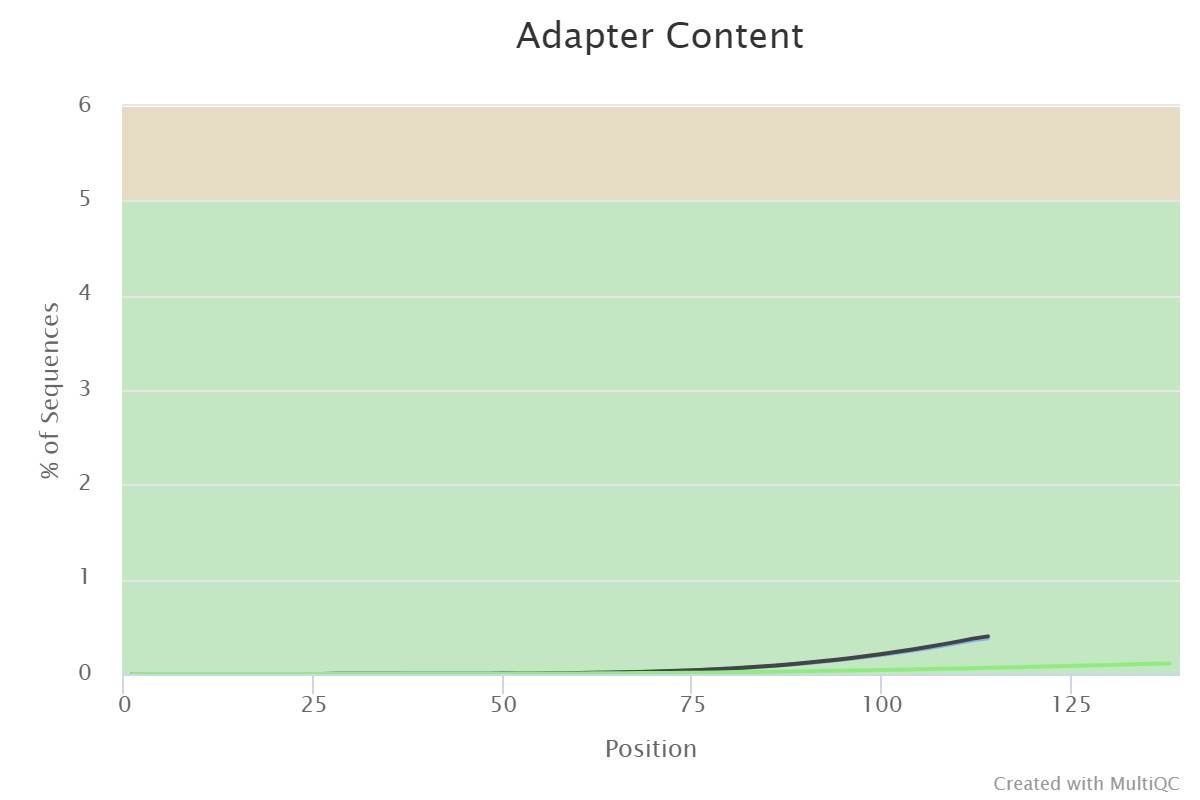

Supplement: Supplementary file 1 [file cimb-45-00365-s001.zip › Figure S4.jpeg]

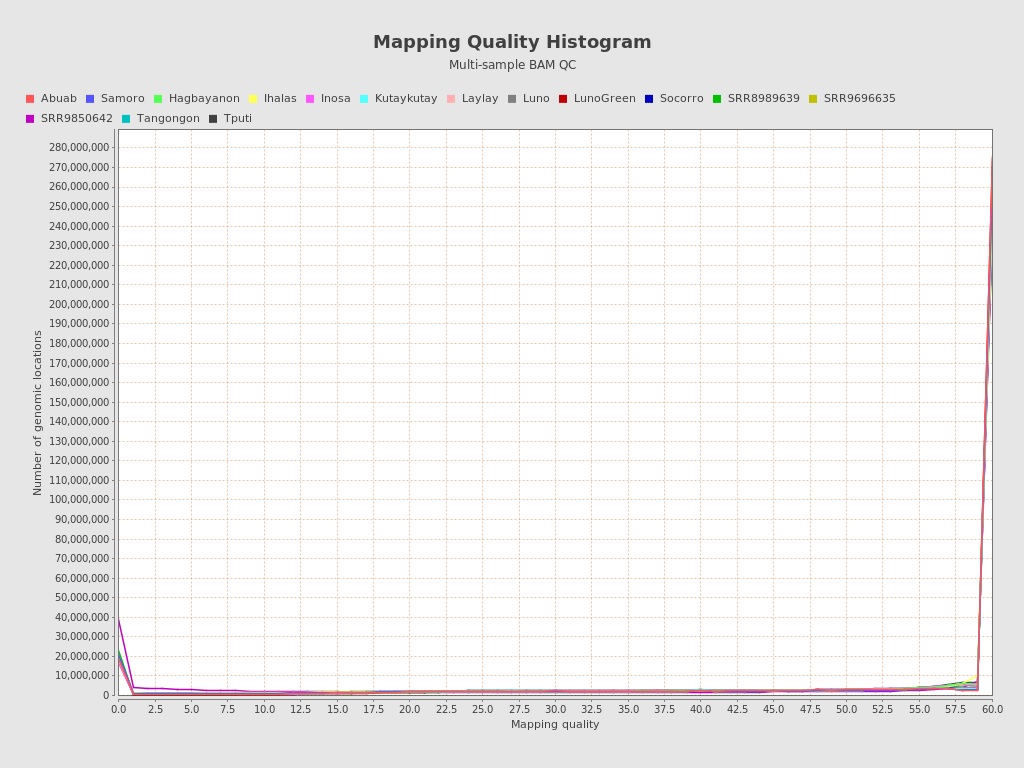

Supplement: Supplementary file 1 [file cimb-45-00365-s001.zip › Figure S6.jpg]
